# Ovarian cancer survival by stage, histotype, and pre-diagnostic lifestyle factors, in the prospective UK Million Women Study

**DOI:** 10.1016/j.canep.2021.102074

**Published:** 2022-02

**Authors:** Kezia Gaitskell, Carol Hermon, Isobel Barnes, Kirstin Pirie, Sarah Floud, Jane Green, Valerie Beral, Gillian K. Reeves, on behalf of the Million Women Study Collaborators

**Affiliations:** a Cancer Epidemiology Unit, Nuffield Department of Population Health, University of Oxford, Oxford, UK; b Nuffield Division of Clinical Laboratory Sciences, Radcliffe Department of Medicine, University of Oxford, Oxford, UK; c Department of Histopathology, John Radcliffe Hospital, Oxford, UK

**Keywords:** 95% CI, 95% Confidence Interval, BMI, Body Mass Index, ICD-10, International Classification of Diseases, version 10, ICD-O, International Classification of Diseases for Oncology, NHS, the UK National Health Service, RR, Relative Risk, SD, Standard Deviation, Ovarian cancer, Survival, Histological type, Stage, Risk factors, Cohort study, Epidemiology

## Abstract

**Background:**

Ovarian cancer is the fifth leading cause of cancer mortality in UK women. Ovarian cancer survival varies by disease stage at diagnosis, but evidence is mixed on the effect of tumour histological type (histotype) and other factors.

**Methods:**

1.3 million UK women completed a detailed health questionnaire in 1996–2001 and were followed for incident cancers and deaths via linkage to national databases. Using Cox regression models, we estimated adjusted relative risks (RRs) of death from ovarian cancer, by stage at diagnosis, tumour histotype, and 16 other personal characteristics of the women.

**Results:**

During 17.7 years’ average follow-up, 13,222 women were diagnosed with ovarian cancer, and 8697 of them died from the disease. Stage at diagnosis was a major determinant of survival (stage IV vs I, RR=10.54, 95% CI: 9.16–12.13). Histotype remained a significant predictor after adjustment for stage and other factors, but associations varied over the follow-up period. Histotype-specific survival was worse for high-grade than low-grade tumours. Survival appeared worse with older age at diagnosis (per 5 years: RR=1.19, 95% CI: 1.15–1.22), higher BMI (per 5-unit increase: RR=1.06, 95% CI: 1.02–1.11), and smoking (current vs never: RR=1.17, 95% CI: 1.07–1.27), but there was little association with 13 other pre-diagnostic reproductive, anthropometric, and lifestyle factors.

**Conclusion:**

Stage at diagnosis is a strong predictor of ovarian cancer survival, but tumour histotype and grade remain predictors of survival even after adjustment for stage and other factors, contributing further evidence of biological dissimilarity between the ovarian cancer histotypes. Obesity and smoking represent potentially-modifiable determinants of survival, but the stronger association with stage suggests that improving earlier diagnosis would have a greater impact on increasing ovarian cancer survival.

## Highlights

• This study investigated ovarian cancer survival in a cohort of 1.3 million UK women.
• After 18 years of follow-up, the study included 13,222 incident ovarian cancers.
• Stage, age and histotype were significantly associated with ovarian cancer survival.
• Survival was also worse with higher BMI and smoking, but with less impact than stage.

## Introduction

Ovarian cancer is the seventh leading cause of cancer mortality in women worldwide, and the fifth leading cause in women in Europe and the USA [1]. National UK statistics report five-year survival for ovarian cancer of 31.0% [2]. This poor survival is partly attributable to late stage at diagnosis: in both UK and US populations, about two-thirds of women have advanced disease (stage III or IV) at diagnosis [3], [4].

There is increasing evidence from histopathological and molecular studies that the different histological types (histotypes) of epithelial ovarian cancer have distinct aetiologies. Many high-grade serous ovarian carcinomas (the most common histotype) are hypothesised to arise from precursor lesions within the fallopian tubal epithelium, while many endometrioid and clear cell carcinomas may originate from endometriosis; the origins of mucinous tumours are still debated [5].

We have previously shown that risk factors for incident ovarian cancer vary by histotype, with heterogeneity in the associations with parity [6], tubal ligation [7], smoking [8], and use of menopausal hormones [9]. Survival might also vary by histological type, but few studies have sufficient cases, and the necessary information on other relevant factors, to explore variation in survival by histotype with adjustment for stage at diagnosis and other potential confounding factors.

A study based on USA cancer registry data [4] has shown a significant association between ovarian cancer histotype and survival, after accounting for stage, as has a study in an Australian cohort [10], but both studies had limited information on other personal characteristics, such as body mass index (BMI), smoking, and reproductive factors. Another USA study, using electronic medical records, reported no association between lifestyle factors and long-term survival in women with ovarian cancer, but information on factors such as BMI and smoking status was missing for half the population, and the analysis was limited to high-grade serous tumours [11]. Others have reported an association between worse survival and pre-diagnosis obesity [12], smoking [13], [14], [15], poor diet [16], and lack of recreational physical activity [17].

We explored the association between ovarian cancer survival and stage at diagnosis, histotype, and reproductive, anthropometric and lifestyle factors, in a national cohort of over 1 million UK women, with over 20 years' follow-up for ovarian cancer incidence and cause-specific mortality.

## Materials and methods

The Million Women Study is a population-based prospective study [18]. Women invited for National Health Service (NHS) breast screening at 66 screening centres in England and Scotland were recruited in 1996–2001 when aged 50–64 years. Participants completed a questionnaire regarding health, sociodemographic, and lifestyle factors. Questionnaires can be viewed at www.millionwomenstudy.org. Information on data access is available at www.millionwomenstudy.org/data_access/.

Follow-up was via record-linkage to routinely-collected NHS data on cancer registrations and deaths from Public Health England and Information Services Division for Scotland. Cancers and causes of death are coded to ICD-10 (International Classification of Diseases, 10th revision) [19]; tumour morphology is coded to the International Classification of Diseases for Oncology, ICD-O [20], [21]. The study was approved by the Oxford and Anglia Multi-Centre Research Ethics Committee (MREC 97/01). Written consent was given at recruitment to consult medical records.

To identify incident ovarian cancers, women were excluded if they had a previous diagnosis of cancer (other than non-melanoma skin cancer) prior to recruitment (n = 39,362), or if they reported previous bilateral oophorectomy at recruitment (n = 105,348).

We defined incident ovarian cancer as a new diagnosis of cancers of the ovary (ICD-10 code C56), fallopian tube (C57), or peritoneum (C48, excluding C48.0, retroperitoneum) occurring after recruitment up to the end of follow-up for cancer incidence (31st December 2018). For histotype analyses, eight main histological groups were derived: serous borderline tumours, serous carcinomas, mucinous borderline tumours, mucinous carcinomas, endometrioid carcinomas, clear cell carcinomas, carcinosarcomas, and other/ unspecified malignant tumours (see Supplementary Table 1 for details).

For some analyses, histotype-specific carcinomas were further divided by grade: serous and mucinous carcinomas were divided into low-grade (grade 1) vs high-grade (grade 2+) [7], [22], [23], [24]; endometrioid carcinomas were divided into low-grade (grade 1–2) and high-grade (grade 3) [25], [26]. Clear cell carcinomas and carcinosarcomas are high grade by definition [22]. For analyses of tumour stage at diagnosis we used International Federation of Gynaecology and Obstetrics (FIGO) stage [27] where available, supplemented by TNM stage [28] where necessary.

Survival in women diagnosed with ovarian cancer was examined in relation to age at diagnosis (50-64, 65–69, ≥70 years), stage at diagnosis (I, II, III, IV), tumour histological type (as detailed above), calendar year of diagnosis (<2005, 2005–2009, ≥2010), pre-diagnostic parity (nulliparous, 1, 2, ≥3 births), oral contraceptive use (never, <5 years, ≥5 years), age at menarche (<12, 12–13, ≥14), tubal ligation (no, yes), hysterectomy (no, yes), menopausal hormone therapy use (never, ever), family history of breast cancer (no, yes), height (<160, 160–164, ≥165 cm), body mass index (<25, 25–29, ≥30 kg/m^2^), educational attainment (none, secondary/technical, tertiary), alcohol intake (none, ≤7 units, >7 units per week), tobacco use (never, past, current), frequency of strenuous exercise (<once, ≥once per week), and tertiles of socioeconomic deprivation based on the Townsend deprivation index [29].

All-cause survival time was calculated from the date of diagnosis to the date of death (from any cause), date of emigration or loss to follow-up, or date of last follow-up for survival (31st December 2019). For ovarian cancer-specific survival time, death was attributed to ovarian cancer if recorded anywhere on the death certificate, or if the underlying cause of death was recorded as a malignant neoplasm of ill-defined, secondary or unspecified site that seemed likely to be ovarian cancer in the context of someone with a known previous diagnosis of ovarian cancer (neoplasm of the pelvis (C76.3); secondary malignant neoplasm of retroperitoneum and peritoneum (C78.6); secondary malignant neoplasm of the ovary (79.6), other specified sites (C79.8), or unspecified site (C79.9); or malignant neoplasm of unspecified site (C80)). Women were censored at death from another cause, or at the end of follow-up, whichever occurred first.

A lifetable approach was used to estimate ovarian cancer-specific survival after 1, 5, and 10 years from diagnosis. We also used Cox proportional hazards models to calculate adjusted hazard ratios (‘relative risks’ (RRs)) and 95% confidence intervals (CIs) of death in those with ovarian cancer. The proportional hazards assumption was assessed using tests based on Schoenfeld residuals [30].

Cox regression models were adjusted for age at diagnosis, year of diagnosis, deprivation, BMI, height, tubal ligation, hysterectomy, use of contraceptive and menopausal hormones, parity, smoking, family history of breast cancer, age at menarche, alcohol intake, frequency of strenuous exercise, and educational attainment, and stratified by geographical region (10 regions based on the breast screening programme recruitment centres), stage at diagnosis, and histological type. Missing data for the adjustment variables (≤6% for each variable) were assigned to a separate category. Regression model analyses were restricted to cases with known stage at diagnosis to ensure adequate adjustment for stage.

STATA version 17 [31] was used for all analyses; figures were plotted in STATA and R [32], [33]. Statistical tests were two-sided, with significance defined as p-value < 0·05.

## Results

During a mean of 17.7 (Standard Deviation (SD) 5.0) years of follow-up of 1,219,603 women, 13,222 women were first diagnosed with ovarian cancer, of whom 8697 (66%) died before 31st December 2019 due to ovarian cancer, and 9307 (70%) died from any cause, after a mean of 4.5 (SD 5.1) years of follow-up from diagnosis. Women who were subsequently diagnosed with ovarian cancer were recruited at a mean age of 56.8 (SD 4.8) and diagnosed with ovarian cancer at a mean age of 67.8 (SD 7.2). 137 women had a date of ovarian cancer diagnosis that coincided with the date of death and so were excluded from subsequent survival analyses, leaving a population of 13,085 cases.

There was little difference in patterns of overall versus ovarian cancer-specific survival (see Supplementary Figure 1); subsequent ovarian cancer survival analyses included only deaths attributed to the disease, with deaths from other causes censored on the date of death. The lifetable estimates for 1-year, 5-year, and 10-year survival were 76% (95% CI: 75–77), 38% (95% CI: 37–39%), and 29% (95% CI: 28–30%), respectively, for deaths from ovarian cancer. For fully-malignant tumours (i.e. excluding borderline tumours), the 1-year, 5-year, and 10-year survival was 74%, 33%, and 23%, respectively (Supplementary Table 2).

The most common tumour histological type was serous carcinoma (46%, n = 6068), including 126 low-grade (grade 1) and 2956 high-grade (grade ≥2) serous carcinomas (grade was missing for 2986 cases). Other tumour types included serous borderline tumours (4%, n = 514), mucinous borderline tumours (5%, n = 617), mucinous carcinomas (4%, n = 577), endometrioid carcinomas (6%, n = 797), clear cell carcinomas (4%, n = 517), and carcinosarcomas (3%, n = 376) (Table 1). 28% of cases were of other or unspecified type (n = 3756), the majority being unspecified carcinomas or adenocarcinomas; non-epithelial tumours accounted for only 1% of cases (n = 149) (Supplementary Table 1).

**Table 1:** Characteristics of Million Women Study participants with ovarian cancer by ovarian cancer-specific survival

	Death from ovarian cancer		
Characteristic	No (n = 4525)	Yes (n = 8697)	All (N = 13,222)
Age at diagnosis, mean (SD)	67.8 (7.3)	67.8 (7.2)	67.8 (7.2)
Follow-up time from diagnosis (years), mean (SD)	8.8 (6.0)	2.2 (2.3)	4.5 (5.1)
**Lifestyle factors**			
Body mass index (kg/m^2^), mean (SD)	26.2 (4.6)	26.4 (4.8)	26.3 (4.7)
Height (cm), mean (SD)	162.4 (6.6)	162.5 (6.6)	162.5 (6.6)
Socioeconomic status, lower third, % (n)	31 (1382)	33 (2817)	32 (4199)
Tertiary education, % (n)	13 (581)	12 (1020)	12 (1601)
Strenuous exercise ≥once/week, % (n)	40 (1735)	40 (3304)	40 (5039)
Alcohol, > 7 units/ week, % (n)	20 (914)	18 (1565)	19 (2479)
Current smoker, % (n)	19 (791)	19 (1528)	19 (2319)
**Reproductive/ hormonal factors**			
Age at menarche, mean (SD)	13.0 (1.6)	13.0 (1.6)	13.0 (1.6)
Nulliparous, % (n)	15 (674)	13 (1142)	14 (1816)
Ever use of oral contraceptive pill, % (n)	56 (2501)	50 (4266)	52 (6767)
Ever use of menopausal hormones, % (n)	48 (2161)	49 (4211)	49 (6372)
Hysterectomy, % (n)	20 (885)	19 (1681)	20 (2566)
Tubal ligation, % (n)	19 (818)	17 (1474)	18 (2292)
Family history of breast cancer	11 (477)	11 (907)	11 (1384)
**Tumour characteristics**			
Histological type, % (n)			
Serous borderline tumour	11 (479)	0.4 (35)	4 (514)
Mucinous borderline tumour	13 (594)	0.3 (23)	5 (617)
Serous carcinoma	36 (1609)	51 (4459)	46 (6068)
Mucinous carcinoma	8 (352)	3 (225)	4 (577)
Endometrioid carcinoma	11 (481)	4 (316)	6 (797)
Clear cell carcinoma	6 (259)	3 (258)	4 (517)
Carcinosarcoma	2 (76)	3 (300)	3 (376)
Other/ Unspecified	15 (675)	35 (3081)	28 (3756)
Stage at diagnosis, % (n)			
Stage I	53 (1558)	6 (295)	24 (1853)
Stage II	10 (287)	5 (228)	7 (515)
Stage III	29 (848)	59 (2858)	47 (3706)
Stage IV	9 (265)	31 (1497)	22 (1762)

Notes: Table shows column %. Numbers may not sum to total due to missing data.

Disease stage, where known, was strongly associated with histotype (Supplementary Table 3). Borderline tumours were (partly by definition) usually diagnosed at low stage; the majority of endometrioid, clear cell, and mucinous carcinomas were diagnosed at stage I+II; the majority of serous carcinomas, carcinosarcomas, and cases of other/ unspecified histological type were diagnosed at stage III+IV. Disease stage at diagnosis was unknown in 41% of cases (n = 5386). Data quality improved substantially over time: information on stage at diagnosis was missing in 65% of cases diagnosed prior to 2005, but in only 12% of cases diagnosed from 2015 onwards, and the proportion of cases of other/ unspecified histotype fell from 33% prior to 2005 to 21% from 2015 onwards. The proportion of cases with unknown grade fell in more recent years for mucinous and endometrioid carcinomas, but increased for serous carcinomas (Supplementary Table 4).

**Fig. 1:**
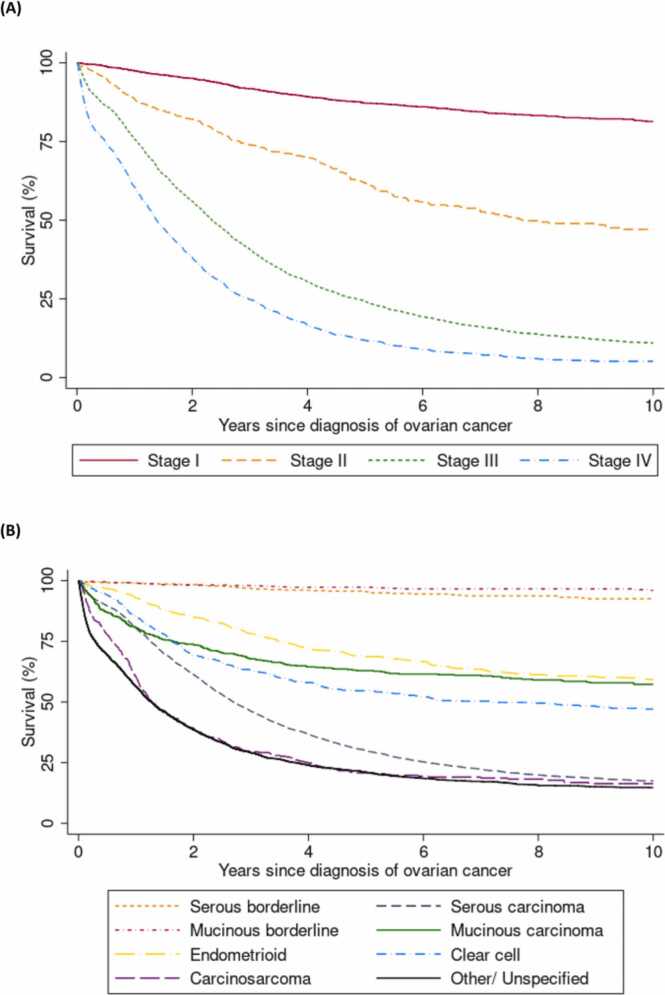
**Ovarian cancer survival by time and (A) Stage at diagnosis and (B) Histological type** This figure shows the Kaplan-Meier survival curves for survival after diagnosis of ovarian cancer, for deaths attributed to ovarian cancer, by (A) stage at diagnosis in women with known stage (N = 7831) and (B) tumour histotype in all women diagnosed with ovarian cancer (N = 13,085). Cases diagnosed at death (n = 137) are excluded.

Fig. 1 shows Kaplan-Meier survival curves, illustrating the variation in ovarian cancer survival by stage (Fig. 1 (A)) and tumour histotype (Fig. 1 (B)). As expected, stage at diagnosis was highly predictive of survival: 5-year survival was 87% for those diagnosed at stage I, 62% for stage II, 26% for stage III, and 14% for stage IV (Fig. 1 (A) and Supplementary Table 2). Tumour histotype also predicted survival, with 5-year survival being excellent for women diagnosed with serous and mucinous borderline tumours (95% and 97% respectively), intermediate for endometrioid (69%), mucinous (63%), and clear cell carcinomas (54%), and poor for serous carcinomas (31%), carcinosarcomas (21%), and tumours of other/ unspecified type (21%) (Fig. 1 (B) and Supplementary Table 2).

Hazards were non-proportional over time for both stage and histotype, largely in the first year after diagnosis, with mucinous and clear cell carcinomas having similar survival to serous carcinomas early on, but better long-term survival, and carcinosarcomas and other/ unspecified tumours having very poor survival in the first year. For example, survival at one year after diagnosis was approximately 80% for both women with serous carcinomas and those with mucinous carcinomas (Supplementary Table 2); however, for those who survived the first year, survival at the end of the second year was 91% for those with mucinous carcinomas and only 76% for those with serous carcinomas (Supplementary Table 5). The poor survival of carcinosarcomas relative to other histotypes was particularly evident in the small proportion of cases diagnosed at Stage I and II (Supplementary Figure 2). Subsequent analyses were thus stratified by (rather than adjusted for) tumour stage and histotype where possible. Analyses of stage and histotype are presented separately for the first year following diagnosis versus after the first year, in addition to an overall average for the entire period of follow-up. As stage was such a strong predictor of survival, regression analyses were restricted to cases with known stage (n = 7831). As deaths from borderline tumours were rare, subsequent regression analyses are restricted to invasive ovarian cancer, unless specifically showing results for tumour histological types.

**Fig. 2:**
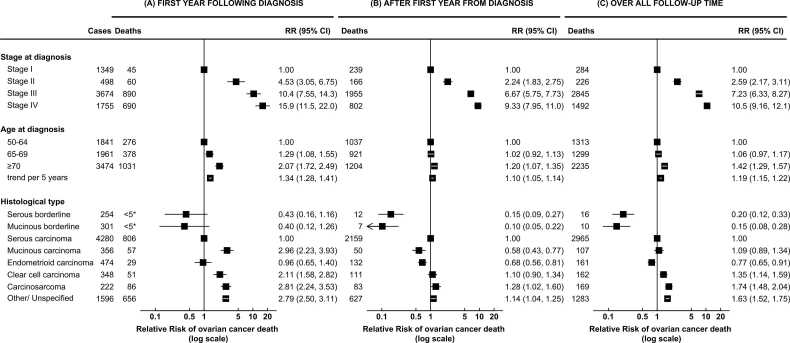
**Ovarian cancer survival by stage at diagnosis, age at diagnosis, and histological type** The figure shows RRs and 95% CI for the association between ovarian cancer survival and stage at diagnosis, age at diagnosis, and histological type, in women with ovarian cancer of known stage at diagnosis (n = 7831). Results are shown separately for the first year following diagnosis (A), after the first year (B), and over all follow-up time (C). Analyses of age and stage at diagnosis are restricted to cases of invasive ovarian cancer (n = 7276, excluding borderline tumours). RRs are adjusted for age at diagnosis, year of diagnosis, deprivation, tubal ligation, hysterectomy, use of contraceptive or menopausal hormones, parity, BMI, smoking, alcohol intake, age at menarche, family history of breast cancer, frequency of strenuous exercise, and education, and stratified by region, disease stage at diagnosis, and tumour histological type, as appropriate. *The precise number of cases is omitted for cells in which n < 5, in accordance with guidance from the Office for National Statistics.

Fig. 2 shows adjusted RRs of death from ovarian cancer by stage, age at diagnosis, and tumour histotype. As expected, higher stage was strongly associated with worse survival, even after adjustment for age at diagnosis, histology, and reproductive, anthropometric and lifestyle characteristics. Compared to women diagnosed at stage I, women diagnosed at stage III had a seven-fold risk of death overall (RR=7.23, 95% CI: 6.33–8.27), and stage IV a ten-fold risk (RR=10.54, 95% CI: 9.16–12.13) (Fig. 2 (C)). This strong association with stage was seen throughout the follow-up period, though the magnitude of the association appeared larger in the first year following diagnosis (Fig. 2 (A) vs (B)).

Older age at diagnosis was also, as expected, associated with poorer survival. Overall, each five-year increase in age was associated with a 19% increase in risk of death from ovarian cancer (RR=1.19, 95% CI: 1.15–1.22), though the magnitude of this association likewise appeared larger in the first year following diagnosis (Fig. 2).

Histological type remained a significant predictor of survival even after adjustment for stage, age at diagnosis, and other factors. However, some associations showed substantial variation over time. Compared to serous carcinomas, women with serous and mucinous borderline tumours and endometrioid carcinomas had substantially decreased adjusted risks of dying overall (serous borderline tumours: RR=0.20, 95% CI: 0.12–0.33; mucinous borderline tumours: RR=0.15, 95% CI: 0.08–0.28; endometrioid carcinomas: RR=0.77, 95% CI: 0.65–0.91), and these patterns were similar throughout follow-up though under-powered in the first year. Women with carcinosarcomas had a significantly increased risk of dying compared to those with serous carcinomas, which was more pronounced in the first year following diagnosis (RR=2.81, 95% CI: 2.24–3.53) than subsequently (RR=1.28, 95% CI: 1.02–1.60).

Mucinous carcinomas and clear cell carcinomas showed different associations in different periods: within the first year from diagnosis, compared to serous carcinomas, women with mucinous carcinomas (RR=2.96, 95% CI: 2.23–3.93) and clear cell carcinomas (RR=2.11, 95% CI: 1.58–2.82) both had significantly increased adjusted risks of dying. However, beyond the first year from diagnosis, the risk of ovarian cancer death compared to serous carcinomas was significantly lower for mucinous carcinomas (RR=0.68, 95% CI: 0.56–0.81), and not significantly different for clear cell carcinomas (RR=1.10, 95% CI: 0.90–1.34).

**Fig. 3:**
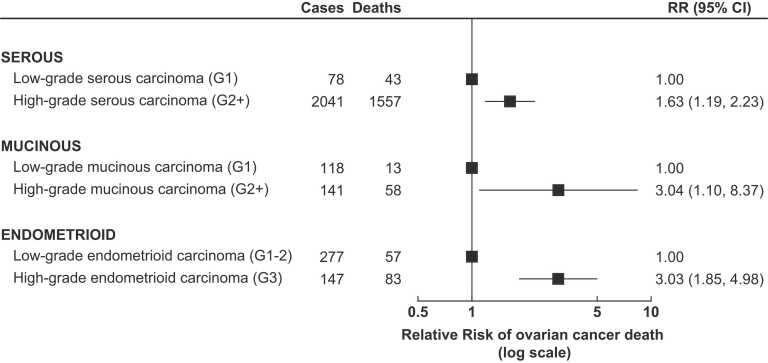
**Ovarian cancer survival by tumour grade** The figure shows RRs and 95% CI for the association between ovarian cancer survival and grade for various histotypes of ovarian carcinoma, in women with ovarian cancer of known stage at diagnosis. RRs are adjusted as appropriate for age at diagnosis, year of diagnosis, deprivation, tubal ligation, hysterectomy, use of contraceptive or menopausal hormones, parity, BMI, smoking, alcohol intake, age at menarche, family history of breast cancer, frequency of strenuous exercise, and education, and stratified by region and stage at diagnosis. Note: Clear cell carcinoma and carcinosarcoma are regarded as high grade by definition, and thus are not shown here.

Fig. 3 shows the association between tumour grade and survival for serous, mucinous, and endometrioid carcinomas. In all cases, high-grade tumours were associated with poorer survival compared to low-grade tumours of the same histological type.

**Fig. 4:**
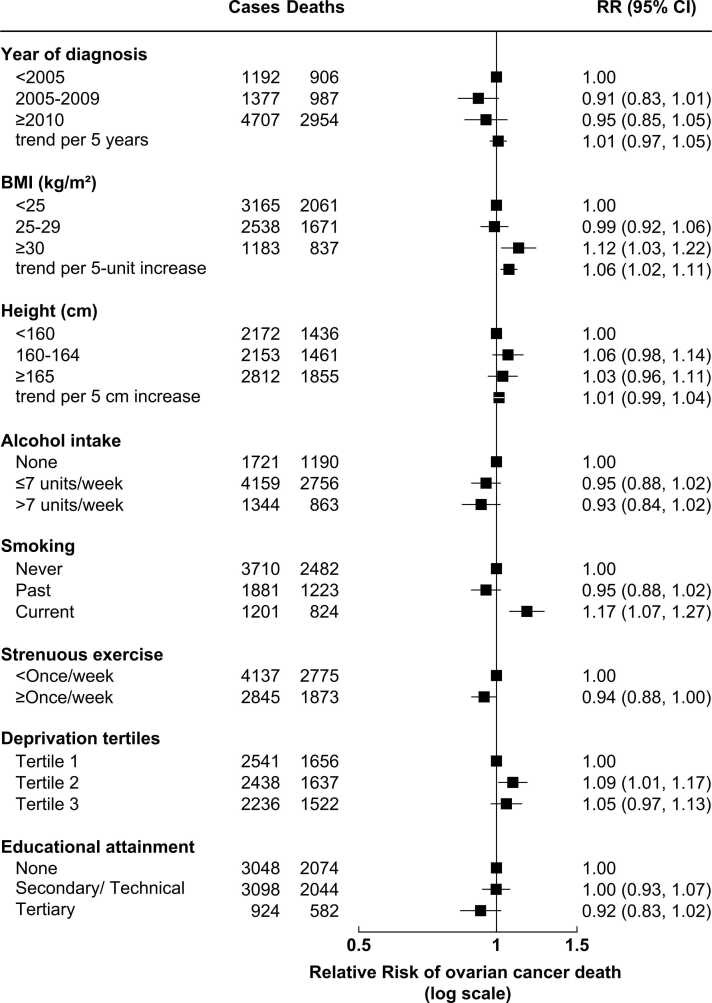
**Ovarian cancer survival by anthropometric and lifestyle factors** The figure shows RRs and 95% CI for the association between ovarian cancer survival and anthropometric and lifestyle factors in women with invasive ovarian cancer of known stage at diagnosis (N = 7276). RRs are adjusted as appropriate for age at diagnosis, year of diagnosis, deprivation, tubal ligation, hysterectomy, use of contraceptive or menopausal hormones, parity, BMI, smoking, alcohol intake, age at menarche, family history of breast cancer, frequency of strenuous exercise, and education, and stratified by region, stage at diagnosis, and tumour histological type. Numbers of cases may not sum to total, due to missing information.

Fig. 4 shows the association between ovarian cancer survival and anthropometric and lifestyle factors. There was some evidence of worse survival with higher pre-diagnostic BMI, with an overall 6% higher risk per 5-unit increase in BMI (RR=1.06, 95% CI: 1.02–1.11). There was also evidence of worse survival in current smokers (RR=1.17, 95% CI: 1.07–1.27 compared to never smokers). There was little or no evidence of an association between ovarian cancer survival and year of diagnosis, height, alcohol intake, strenuous exercise, deprivation, or educational attainment.

There was no evidence that the associations between BMI or smoking and ovarian cancer survival varied significantly by tumour histological type (heterogeneity: BMI, p = 0.9; smoking, p = 0.6). The association between BMI and ovarian cancer was only statistically significant for serous carcinoma (per 5-unit increase in BMI, RR=1.06, 95% CI: 1.01–1.12); the greatest magnitude of relative risk was seen with mucinous carcinomas, but this was not statistically significant (RR=1.26, 95% CI: 0.94–1.69) (Supplementary Figure 3). The association between current smoking and poorer ovarian cancer survival was not statistically significant for any individual tumour histological type, but analyses were under-powered (Supplementary Figure 4).

**Fig. 5:**
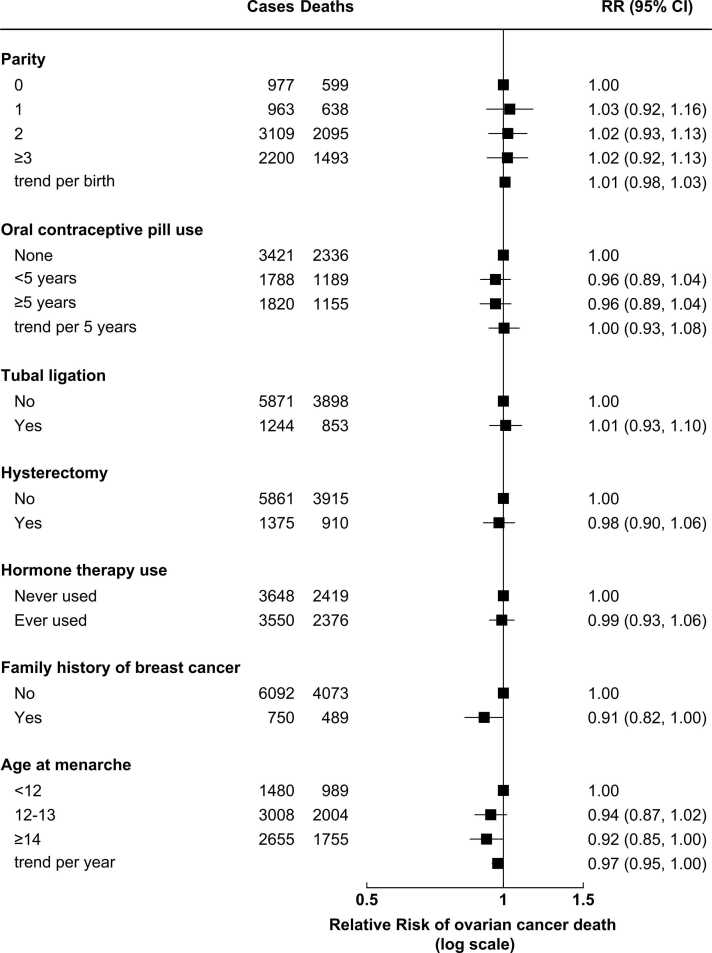
**Ovarian cancer survival by reproductive and hormonal factors** The figure shows RRs and 95% CI for the association between ovarian cancer survival and reproductive and hormonal factors in women with invasive ovarian cancer of known stage at diagnosis (N = 7276). RRs are adjusted as appropriate for age at diagnosis, year of diagnosis, deprivation, tubal ligation, hysterectomy, use of contraceptive or menopausal hormones, parity, BMI, smoking, alcohol intake, age at menarche, family history of breast cancer, frequency of strenuous exercise, and education, and stratified by region, stage at diagnosis, and tumour histological type. Numbers of cases may not sum to total, due to missing information.

Fig. 5 shows the association between ovarian cancer survival and reproductive/ hormonal factors. There was little or no evidence of an association between ovarian cancer survival and parity, use of the oral contraceptive pill, tubal ligation, hysterectomy, use of menopausal hormone therapy, family history of breast cancer, or age at menarche.

## Discussion

In this large prospective study of 13,222 women with ovarian cancer and information on both tumour and pre-diagnostic personal characteristics, stage at diagnosis was strongly associated with survival, as expected. After adjustment for stage and other factors, age at diagnosis had a modest association. Tumour histotype was also significantly associated with survival, even after adjustment for stage and other factors: compared to serous carcinomas, serous and mucinous borderline tumours had much better survival, endometrioid carcinomas moderately better survival, and carcinosarcomas worse survival, consistent with other studies [4], [10]. Associations with mucinous and clear cell carcinomas were complex, with different associations seen in the first year after diagnosis compared to subsequent years, in keeping with other studies [4].

One possible explanation for this difference might be variation by histotype in responsiveness to treatment [4]. Ovarian cancer is typically treated with cytoreductive surgery and combined platinum and taxane-based chemotherapy [34]. Many high-grade serous ovarian carcinomas initially respond well to chemotherapy but later develop platinum resistance; by contrast, other histotypes (such as mucinous and clear cell carcinomas) tend to be insensitive to platinum-based chemotherapy [35]. In addition, mucinous and clear cell carcinomas diagnosed at high stage have particularly poor prognosis in the first few years, as seen here and previously reported in other populations [4]. Thus, serous carcinomas may initially have a better survival than some other histotypes due to good chemotherapy response in serous carcinomas and poor survival of other histotypes at high stage, but then a worse survival in later years as chemotherapy resistance develops. Adjustment for stage was important, as histotype was strongly associated with stage at diagnosis.

For those serous, endometrioid, and mucinous carcinomas with grade information, higher grade was associated with worse survival after adjustment for stage. As low-grade and high-grade ovarian serous carcinomas are now thought to be different diseases, with distinct aetiologies and precursor lesions, rather than simply grades within a single disease process [22], other recent studies have often looked at survival separately for high-grade versus low-grade serous carcinomas, but have not examined the association between tumour grade and survival for other types of ovarian cancer [4]. Our results are thus novel and show apparently strong associations between tumour grade and survival, even after adjustment for other factors.

We found that higher BMI was associated with a modest but statistically significant worsening of ovarian cancer survival, in line with findings from previous studies including a pooled analysis of retrospective studies [12]. We also found that smoking was associated with worse survival, consistent with previous reports including a pooled analysis of retrospective studies [13] and the prospective Nurses’ Health Study [15]. We did not find that these associations varied by tumour histological type, but this might be due to insufficient statistical power.

Several other studies have suggested that menopausal hormone use may be associated with improved ovarian cancer survival [36], [37], [38], [39], but we found no such association, nor did the NIH-AARP study [40]. We found little or no evidence of an association between ovarian cancer survival and other reproductive or hormonal factors, broadly in keeping with previous reports [37]. Other studies have also reported worse survival associated with pre-diagnostic physical inactivity [17], but this was not evident in our data, nor in the Nurses’ Health Study [41].

This analysis represents one of the largest studies of ovarian cancer survival with prospective information on reproductive, lifestyle, and anthropometric factors, in addition to stage at diagnosis, tumour histotype and grade.

Strengths of this study include the population-based nature of the original sample, the prospective collection of information on anthropometric, reproductive, and lifestyle factors (avoiding recall bias), and the almost-complete follow-up for cancer diagnosis and mortality (only 1% of the original cohort have been lost to follow-up).

Limitations included our reliance on registry information on tumour histotype, and incomplete information on tumour stage and grade. Changes in the classification of ovarian cancer over time also make the interpretation of historically-coded registry data more challenging. For example, guidance on the assignment of primary cancer site (to ovary, fallopian tube, or peritoneum) have changed substantially [42]; we dealt with this by including cancers of all three sites as ‘ovarian’ cancer. Changes in understanding of the pathogenesis of serous ovarian cancer have also led to the most recent classification assigning separate codes for low-grade versus high-grade serous carcinomas [43], which might explain the observed fall in the proportion of serous carcinomas with additional grade information. We also had little information on treatment including surgical debulking status, extent of residual disease, or details of chemotherapy given, and thus could not adjust for these factors or explore their association with survival. The age profile of our cohort (almost all participants were aged 50 or over at recruitment) also meant that we were unable to investigate survival in younger women, and had few cases of tumours more common in younger women (e.g. germ cell tumours or sex cord-stromal tumours).

In conclusion, stage at diagnosis is a strong predictor of ovarian cancer survival, but tumour histotype and grade remain predictors of survival even after adjustment for stage and other factors. This is consistent with increasing evidence of the biological dissimilarities between the ovarian cancer histotypes, in terms of both aetiology and prognosis. We also found that higher BMI, and current smoking, were associated with worse survival – corroborating associations previously reported in retrospective studies. However, we found little or no evidence of an association with other pre-diagnostic anthropometric, reproductive and lifestyle factors. If the associations seen with BMI and smoking were causal, these might represent potentially-modifiable means of improving survival in ovarian cancer, which is often poor. However, the associations seen with these lifestyle factors are of much smaller magnitude than those seen with stage at diagnosis – and so interventions aimed at improving early diagnosis of ovarian cancer are likely to have greater impact on improving survival.

## Ethical approval

The Million Women Study received ethical approval from the Oxford and Anglia Multi-Centre Research Ethics Committee (MREC 97/01). All participants provided written consent at recruitment.

## Authorship contribution statement

KG and CH wrote the manuscript with support from IB, KP, SF, JG, VB and GKR. All authors made substantial contributions to the interpretation of the findings, contributed to drafting the manuscript or revising it critically for important intellectual content, and approved the final version submitted.

## Conflicts of interest

The authors declare no potential conflicts of interest.

## Data Availability

Information on data access for the Million Women Study is available at www.millionwomenstudy.org/data_access/.
