# Supplementary material for: Ovarian cancer survival by stage, histotype, and pre-diagnostic lifestyle factors, in the prospective UK Million Women Study
Source: Cancer Epidemiol. 2022 Feb;76:102074. doi: 10.1016/j.canep.2021.102074 (PMC8785125; doi:10.1016/j.canep.2021.102074)
Supplement: Supplementary Table 1 — Supplementary material [file mmc1.pdf]

Ovarian cancer survival by stage, histotype, and pre-diagnostic lifestyle factors, in the prospective UK Million Women Study

SUPPLEMENTARY MATERIAL

Contents

Supplementary Tables 2

Supplementary Table 1: Classification of tumour histological type 2

Supplementary Table 2: 1, 5, and 10-year ovarian cancer survival, overall and by tumour stage at diagnosis and histological type 9

Supplementary Table 3: Histological type and stage at diagnosis of ovarian cancer cases 10

Supplementary Table 4: Tumour characteristics and missing data, by year of diagnosis 11

Supplementary Table 5: Additional 1, 5, and 10-year ovarian cancer survival, overall and by tumour stage at diagnosis and histological type, for women who survived the first year after ovarian cancer diagnosis 12

Supplementary Figures 13

Supplementary Figure 1: Survival by time since diagnosis of ovarian cancer 13

Supplementary Figure 2: Ovarian cancer survival by time and histological type in cases diagnosed at (A) Stage I and II and (B) Stage III and IV 14

Supplementary Figure 3: The association between BMI (per 5-unit increase) and ovarian cancer survival, by tumour histological type 15

Supplementary Figure 4: The association between smoking and ovarian cancer survival, by tumour histological type 16

Supplementary Tables

Supplementary Table 1: Classification of tumour histological type

The table shows all ICD-O (International Classification of Diseases for Oncology) codes for ovarian cancer in this study (N=13,222), with the corresponding text diagnoses from ICD-O-2 and three editions of ICD-O-3 (with publication year), and the groupings using in this study. As the number of cases of non-epithelial tumours and unspecified malignant tumours were small, they were grouped with other/ unspecified carcinomas for analysis. Abbreviations: NOS, Not Otherwise Specified. *The precise number of cases is omitted for cells in which n<5, in accordance with guidance from the Office for National Statistics.

ICD-O code	Cases	ICD-O-2 (1990)	ICD-O-3 (2000)	ICD-O-3.1 (2013)	ICD-O-3.2 (2020)	Comment
**EPITHELIAL TUMOURS**						
**Serous borderline tumours (n=514)**						
84411	<5*	Code not listed	Code not listed	Code not listed	Code not listed	Code not listed in any version. Code indicates Serous tumour with borderline suffix, so assumed to be serous borderline tumour.
84421	17	Code not listed	Serous cystadenoma, borderline malignancy	Serous cystadenoma, borderline malignancy	Serous borderline tumour, NOS	
84423	357	Serous cystadenoma, borderline malignancy	Code not listed	Code not listed	Code not listed	
84425	12	Code not listed	Code not listed	Code not listed	Code not listed	Code not listed in any version. Closest code is serous cystadenoma of borderline malignancy, so grouped together.
84513	12	Papillary cystadenoma, borderline malignancy	Code not listed	Code not listed	Code not listed	
84611	<5*	Code not listed	Code not listed	Code not listed	Code not listed	Code not listed in any version. Code indicates Serous tumour with borderline suffix, so assumed to be serous borderline tumour.
84621	6	Code not listed	Serous papillary cystic tumour of borderline malignancy	Serous papillary cystic tumour of borderline malignancy	Code not listed	
84623	104	Papillary serous cystadenoma, borderline malignancy	Code not listed	Code not listed	Code not listed	
84625	<5*	Code not listed	Code not listed	Code not listed	Code not listed	

**Serous carcinomas (n=6068)**						
84413	3,071	Serous cystadenocarcinoma NOS	Serous cystadenocarcinoma NOS	Serous cystadenocarcinoma, NOS	Serous carcinoma, NOS	
84415	<5*	Code not listed	Code not listed	Code not listed	Code not listed	Code not listed in any version. Closest code is Serous carcinoma, so grouped together.
84503	37	Papillary cystadenocarcinoma NOS	Papillary cystadenocarcinoma NOS	Papillary cystadenocarcinoma, NOS	Papillary cystadenocarcinoma, NOS	
84603	1,585	Papillary serous cystadenocarcinoma	Papillary serous cystadenocarcinoma	Papillary serous cystadenocarcinoma	Low grade serous carcinoma	
84609	<5*	Code not listed	Code not listed	Code not listed	Code not listed	Code not listed in any version. Closest code is Serous carcinoma, so grouped together.
84613	1,364	Serous surface papillary carcinoma	Serous surface papillary carcinoma	Serous surface papillary carcinoma	High grade serous carcinoma	
90143	7	Code not listed	Serous adenocarcinofibroma	Serous adenocarcinofibroma	Serous adenocarcinofibroma	
**Mucinous borderline tumours (n=617)**						
84701	<5*	Code not listed	Mucinous cystic tumour with moderate dysplasia	Code not listed	Code not listed	
84711	<5*	Code not listed	Code not listed	Code not listed	Code not listed	Code not listed in any version. Code indicates mucinous tumour, with a borderline suffix, so assumed to be mucinous borderline tumour.
84721	40	Code not listed	Mucinous cystic tumour of borderline malignancy	Mucinous cystic tumour of borderline malignancy	Mucinous cystic tumour of borderline malignancy	
84723	545	Mucinous cystadenoma, borderline malignancy	Code not listed	Code not listed	Code not listed	
84725	8	Code not listed	Code not listed	Code not listed	Code not listed	Code not listed in any version. Closest code is for a mucinous borderline tumour, so grouped together.
84733	21	Papillary mucinous cystadenoma, borderline malignancy	Code not listed	Code not listed	Code not listed	
84735	<5*	Code not listed	Code not listed	Code not listed	Code not listed	Code not listed in any version. Closest code is for a mucinous borderline tumour, so grouped together.

**Mucinous carcinomas (n=577)**						
84703	235	Mucinous cystadenocarcinoma NOS	Mucinous cystadenocarcinoma NOS	Mucinous cystadenocarcinoma, NOS	Mucinous cystadenocarcinoma, NOS	
84713	12	Papillary mucinous cystadenocarcinoma	Papillary mucinous cystadenocarcinoma	Papillary mucinous cystadenocarcinoma	Code not listed	
84803	296	Mucinous adenocarcinoma	Mucinous adenocarcinoma	Mucinous adenocarcinoma	Mucinous adenocarcinoma	
84805	<5*	Code not listed	Code not listed	Code not listed	Code not listed	Code not listed in any version. Closest code is for a mucinous carcinoma, so grouped together.
84813	22	Mucin-producing adenocarcinoma	Mucin-producing adenocarcinoma	Mucin-producing adenocarcinoma	Mucin-producing adenocarcinoma	
84903	10	Signet ring cell carcinoma	Signet ring cell carcinoma	Signet ring cell carcinoma	Signet ring cell carcinoma	
**Endometrioid carcinomas (n=797)**						
83803	787	Endometrioid carcinoma	Endometrioid adenocarcinoma NOS	Endometrioid adenocarcinoma NOS	Endometrioid adenocarcinoma, NOS	
83813	<5*	Endometrioid adenofibroma, malignant	Endometrioid adenofibroma, malignant	Endometrioid adenofibroma, malignant	Endometrioid adenofibroma, malignant	
83815	<5*	Code not listed	Code not listed	Code not listed	Code not listed	Code not listed in any version. Closest code is for a malignant endometrioid adenofibroma, so grouped together.
85703	<5*	Adenocarcinoma with squamous metaplasia	Adenocarcinoma with squamous metaplasia	Adenocarcinoma with squamous metaplasia	Adenocarcinoma with squamous metaplasia	
**Clear cell carcinomas (n=517)**						
83103	515	Clear cell adenocarcinoma NOS	Clear cell adenocarcinoma NOS	Clear cell adenocarcinoma, NOS	Clear cell adenocarcinoma, NOS	
83133	<5*	Code not listed	Clear cell adenocarcinofibroma	Clear cell adenocarcinofibroma	Clear cell adenocarcinofibroma	

Carcinosarcomas (n=376)						
89403	<5*	Mixed tumour, malignant NOS	Mixed tumour, malignant NOS	Mixed tumour, malignant, NOS	Mixed tumour, malignant, NOS	
89503	138	Mullerian mixed tumour	Mullerian mixed tumour	Mullerian mixed tumour	Mullerian mixed tumour	
89513	18	Mesodermal mixed tumour	Mesodermal mixed tumour	Mesodermal mixed tumour	Mesodermal mixed tumour	
89803	217	Carcinosarcoma NOS	Carcinosarcoma NOS	Carcinosarcoma, NOS	Carcinosarcoma, NOS	
Other/ unspecified carcinomas (n=3156)						
80103	747	Carcinoma NOS	Carcinoma NOS	Carcinoma NOS	Carcinoma, NOS	
80105	<5*	Code not listed	Code not listed	Code not listed	Code not listed	Code not listed in any version. Closest code is for Carcinoma NOS, so grouped together.
80203	49	Carcinoma, undifferentiated NOS	Carcinoma, undifferentiated NOS	Carcinoma, undifferentiated, NOS	Carcinoma, undifferentiated, NOS	
80209	<5*	Code not listed	Code not listed	Code not listed	Code not listed	Code not listed in any version. Closest code is for Carcinoma, undifferentiated NOS, so grouped together.
80213	9	Carcinoma, anaplastic type NOS	Carcinoma, anaplastic, NOS	Carcinoma, anaplastic, NOS	Carcinoma, anaplastic, NOS	
80223	<5*	Pleomorphic carcinoma	Pleomorphic carcinoma	Pleomorphic carcinoma	Pleomorphic carcinoma	
80413	16	Small cell carcinoma NOS	Small cell carcinoma NOS	Small cell carcinoma, NOS	Small cell carcinoma, NOS	
80503	33	Papillary carcinoma NOS	Papillary carcinoma NOS	Papillary carcinoma, NOS	Papillary carcinoma, NOS	
80523	<5*	Papillary squamous cell carcinoma	Papillary squamous cell carcinoma	Papillary squamous cell carcinoma	Papillary squamous cell carcinoma	
80703	31	Squamous cell carcinoma NOS	Squamous cell carcinoma NOS	Squamous cell carcinoma, NOS	Squamous cell carcinoma, NOS	
80713	<5*	Squamous cell carcinoma, keratinising NOS	Squamous cell carcinoma, keratinising NOS	Squamous cell carcinoma, keratinizing, NOS	Squamous cell carcinoma, keratinizing, NOS	
80723	<5*	Squamous cell carcinoma, large cell, non-keratinising	Squamous cell carcinoma, large cell, non-keratinising, NOS	Squamous cell carcinoma, large cell, nonkeratinizing, NOS	Squamous cell carcinoma, large cell, nonkeratinizing, NOS	

81203	6	Transitional cell carcinoma NOS	Transitional cell carcinoma NOS	Transitional cell carcinoma, NOS	Transitional cell carcinoma, NOS	
81403	1,965	Adenocarcinoma NOS	Adenocarcinoma NOS	Adenocarcinoma, NOS	Adenocarcinoma, NOS	
81409	<5*	Code not listed	Code not listed	Code not listed	Code not listed	Code not listed in any version. Closest code is for Adenocarcinoma NOS, so grouped together.
81443	<5*	Adenocarcinoma, intestinal type	Adenocarcinoma, intestinal type	Adenocarcinoma, intestinal type	Adenocarcinoma, intestinal type	
81453	<5*	Carcinoma, diffuse type	Carcinoma, diffuse type	Carcinoma, diffuse type	Carcinoma, diffuse type	
82303	<5*	Solid carcinoma NOS	Solid carcinoma NOS	Solid carcinoma, NOS	Solid carcinoma, NOS	
82403	29	Carcinoid tumour NOS (except of appendix M8240/1)	Carcinoid tumour, NOS	Carcinoid tumour, NOS	Neuroendocrine tumour, NOS	
82433	<5*	Goblet cell carcinoid	Goblet cell carcinoid	Goblet cell carcinoid	Goblet cell carcinoid	
82443	<5*	Composite carcinoid	Composite carcinoid	Mixed neuroendocrine carcinoma	Mixed adenoneuroendocrine carcinoma	
82463	23	Neuroendocrine carcinoma	Neuroendocrine carcinoma, NOS	Neuroendocrine carcinoma, NOS	Neuroendocrine carcinoma, NOS	
82553	<5*	Code not listed	Adenocarcinoma with mixed subtypes	Adenocarcinoma with mixed subtypes	Adenocarcinoma with mixed subtypes	
82603	138	Papillary adenocarcinoma NOS	Papillary adenocarcinoma NOS	Papillary adenocarcinoma, NOS	Papillary adenocarcinoma, NOS	
82903	<5*	Oxyphilic adenocarcinoma	Oxyphilic adenocarcinoma	Oxyphilic adenocarcinoma	Oxyphilic adenocarcinoma	
83203	<5*	Granular cell carcinoma	Granular cell carcinoma	Granular cell carcinoma	Granular cell carcinoma	
83233	23	Mixed cell adenocarcinoma	Mixed cell adenocarcinoma	Mixed cell adenocarcinoma	Mixed cell adenocarcinoma	
83403	<5*	Papillary carcinoma, follicular variant	Papillary carcinoma, follicular variant	Papillary carcinoma, follicular variant	Papillary carcinoma, follicular variant	
84013	<5*	Apocrine adenocarcinoma	Apocrine adenocarcinoma	Apocrine adenocarcinoma	Apocrine adenocarcinoma	
84403	49	Cystadenocarcinoma NOS	Cystadenocarcinoma NOS	Cystadenocarcinoma, NOS	Cystadenocarcinoma, NOS	
85503	<5*	Acinar cell carcinoma	Acinar cell carcinoma	Acinar cell carcinoma	Acinar cell carcinoma	
85603	<5*	Adenosquamous carcinoma	Adenosquamous carcinoma	Adenosquamous carcinoma	Adenosquamous carcinoma	
90003	8	Brenner tumour, malignant	Brenner tumour, malignant	Brenner tumour, malignant	Brenner tumour, malignant	

NON-EPITHELIAL TUMOURS (n=149)						
85903	<5*	Code not listed	Code not listed	Code not listed	Code not listed	Code not listed in any version. Closest code is for Sex cord-gonadal stromal tumour, NOS (85901), so grouped together.
86203	61	Granulosa cell tumour, malignant	Granulosa cell tumour, malignant	Granulosa cell tumour, malignant	Adult granulosa cell tumour of ovary	
86313	<5*	Code not listed	Sertoli-Leydig cell tumour, poorly differentiated	Sertoli-Leydig cell tumour, poorly-differentiated	Sertoli-Leydig cell tumour, poorly differentiated	
86503	<5*	Leydig cell tumour, malignant	Leydig cell tumour, malignant	Leydig cell tumour-malignant	Leydig cell tumour, malignant	
87213	<5*	Nodular melanoma	Nodular melanoma	Nodular melanoma	Nodular melanoma	
88003	8	Sarcoma NOS	Sarcoma NOS	Sarcoma, NOS	Sarcoma, NOS	
88033	<5*	Small cell sarcoma	Small cell sarcoma	Small cell sarcoma	Small cell sarcoma	
88103	9	Fibrosarcoma NOS	Fibrosarcoma NOS	Fibrosarcoma, NOS	Fibrosarcoma, NOS	
88503	<5*	Liposarcoma NOS	Liposarcoma NOS	Liposarcoma, NOS	Liposarcoma, NOS	
88513	<5*	Liposarcoma, well differentiated	Liposarcoma, well differentiated	Liposarcoma, well differentiated	Liposarcoma, well differentiated, NOS	
88543	<5*	Pleomorphic liposarcoma	Pleomorphic liposarcoma	Pleomorphic liposarcoma	Pleomorphic liposarcoma	
88903	31	Leiomyosarcoma NOS	Leiomyosarcoma NOS	Leiomyosarcoma, NOS	Leiomyosarcoma, NOS	
89003	<5*	Rhabdomyosarcoma NOS	Rhabdomyosarcoma NOS	Rhabdomyosarcoma, NOS	Rhabdomyosarcoma, NOS	
89333	<5*	Adenosarcoma	Adenosarcoma	Adenosarcoma	Adenosarcoma	
89363	<5*	Code not listed	Gastrointestinal stromal sarcoma	Gastrointestinal stromal sarcoma	Gastrointestinal stromal tumour	
89633	<5*	Rhabdoid sarcoma	Malignant rhabdoid tumour	Malignant rhabdoid tumour	Rhabdoid tumour, NOS	
89903	5	Mesenchymoma, malignant	Mesenchymoma, malignant	Mesenchymoma, malignant	Mesenchymoma, malignant	
90643	<5*	Germinoma	Germinoma	Germinoma	Germinoma	
90713	<5*	Endodermal sinus tumour	Yolk sac tumour	Yolk sac tumour	Yolk sac tumour, NOS	
90803	<5*	Teratoma, malignant NOS	Teratoma, malignant NOS	Teratoma, malignant, NOS	Teratoma, malignant, NOS	

90843	<5*	Dermoid cyst with malignant transformation	Teratoma with malignant transformation	Teratoma with malignant transformation	Teratoma with malignant transformation	
90903	5	Struma ovarii, malignant	Struma ovarii, malignant	Struma ovarii, malignant	Struma ovarii, malignant	
91103	<5*	Mesonephroma, malignant	Mesonephroma, malignant	Mesonephroma, malignant	Mesonephroma, malignant	
93643	<5*	Peripheral neuroectodermal tumour	Peripheral neuroectodermal tumour	Peripheral neuroectodermal tumour	Ewing sarcoma	
**MALIGNANT TUMOURS NOS (n=451)**						
80003	443	Neoplasm, malignant	Neoplasm, malignant	Neoplasm, malignant	Neoplasm, malignant	
80013	6	Tumour cells, malignant	Tumour cells, malignant	Tumour cells, malignant	Tumour cells, malignant	
80043	<5*	Malignant tumour, fusiform cell type	Malignant tumour, spindle cell type	Malignant tumour, spindle cell type	Malignant tumour, spindle cell type	

References

ICD-O-2 (1990): C. Percy, V. Van Holten, C.S. Muir, International Classification of Diseases for Oncology, 2nd Edition (ICD-O-2), World Health Organization, Geneva, 1990.

ICD-O-3 (2000): A. Fritz, C. Percy, A. Jack, K. Shanmugaratnam, L. Sobin, D. Parkin, S. Whelan, International Classification of Diseases for Oncology, 3rd Edition (ICD-O-3), World Health Organization, Geneva, 2000.

ICD-O-3.1 (2013): A. Fritz, C. Percy, A. Jack, K. Shanmugaratnam, L. Sobin, D. Parkin, S. Whelan, International Classification of Diseases for Oncology, 3rd edition, 1st revision (ICD-O-3.1), World Health Organization, Geneva, 2013.

ICD-O-3.2 (2020): A. Fritz, C. Percy, A. Jack, K. Shanmugaratnam, L. Sobin, D. Parkin, S. Whelan, International Classification of Diseases for Oncology, 3rd Edition, 2nd Revision (ICD-O-3.2), 2020. http://www.iacr.com.fr/images/Newsflash/ICD-O-3.2_final_update09102020.xls.

Supplementary Table 2: 1, 5, and 10-year ovarian cancer survival, overall and by tumour stage at diagnosis and histological type

Ovarian cancer type	Cases	% survival at timepoint (95% CI) 1 year	5 years	10 years
All ovarian cancer	13,085	76% (75-77)	38% (37-39)	29% (28-30)
Fully-malignant ovarian cancer (excluding borderline tumours)	11,954	74% (73-74)	33% (32-34)	23% (22-24)
**Stage at diagnosis**				
Stage I	1853	97% (97-98)	87% (86-89)	81% (79-83)
Stage II	515	88% (85-91)	62% (58-67)	47% (42-52)
Stage III	3705	76% (74-77)	26% (24-27)	12% (11-14)
Stage IV	1758	61% (58-63)	14% (13-16)	7% (5-8)
Unknown	5254	72% (71-73)	36% (35-38)	28% (27-30)
**Ovarian cancer histotypes**				
Serous borderline tumour	514	99% (98-100)	95% (93-97)	92% (89-95)
Mucinous borderline tumour	617	99% (98-100)	97% (95-98)	96% (94-97)
Serous carcinoma	6060	80% (79-81)	31% (30-32)	18% (17-19)
Mucinous carcinoma	575	81% (77-84)	63% (59-67)	57% (53-61)
Endometrioid carcinoma	797	93% (91-94)	69% (65-72)	59% (55-63)
Clear cell carcinoma	517	85% (82-88)	54% (50-59)	47% (42-51)
Carcinosarcoma	376	60% (55-65)	21% (17-25)	17% (13-21)
Other/ Unspecified	3629	56% (55-58)	21% (20-23)	15% (13-16)

Note: Table shows ovarian cancer-specific survival at 1, 5, and 10 years from diagnosis, calculated using a lifetable approach, for all Million Women Study participants diagnosed with ovarian cancer, after exclusion of 137 cases diagnosed at death (N= 13,085). The above crude survival data is unadjusted for age, stage, or other factors.

Supplementary Table 3: Histological type and stage at diagnosis of ovarian cancer cases

Ovarian cancer: Histological type	Stage I	Stage II	Stage III	Stage IV	Total, n
Serous borderline tumour, % (n)	84 (214)	6 (14)	10 (26)	0 (0)	254
Mucinous borderline tumour, % (n)	96 (290)	1 (<5*)	2 (5)	1 (<5*)	301
Serous carcinoma, % (n)	9 (396)	6 (269)	61 (2613)	23 (1002)	4280
Mucinous carcinoma, % (n)	69 (247)	5 (17)	18 (64)	8 (28)	356
Endometrioid carcinoma, % (n)	58 (277)	16 (76)	20 (94)	6 (27)	474
Clear cell carcinoma, % (n)	59 (205)	12 (40)	23 (80)	7 (23)	348
Carcinosarcoma, % (n)	14 (30)	13 (29)	60 (133)	14 (30)	222
Other / Unspecified, % (n)	12 (194)	4 (67)	43 (690)	40 (645)	1596
Total, % (n)	24 (1853)	7 (515)	47 (3705)	22 (1758)	7831

Note: Cases with unknown stage at diagnosis (n=5386), and cases diagnosed at death (n=137) have been excluded. Table shows row %. * The precise number of cases is omitted for cells in which n<5, in accordance with guidance from the Office for National Statistics.

Supplementary Table 4: Tumour characteristics and missing data, by year of diagnosis

Tumour characteristic	<2005	2005-2009	2010-2014	2015+	Total
**Stage, % (n)**					
Stage I	10 (348)	13 (421)	18 (645)	16 (439)	14 (1853)
Stage II	3 (91)	3 (107)	5 (165)	6 (152)	4 (515)
Stage III	16 (590)	21 (702)	35 (1230)	43 (1184)	28 (3706)
Stage IV	7 (243)	7 (250)	18 (625)	23 (644)	13 (1762)
Unknown	65 (2329)	56 (1870)	24 (851)	12 (336)	41 (5386)
**Tumour histological type, % (n)**					
Serous borderline tumour	4 (157)	4 (150)	4 (132)	3 (75)	4 (514)
Mucinous borderline tumour	5 (195)	5 (164)	4 (147)	4 (111)	5 (617)
Serous carcinoma	34 (1234)	41 (1380)	51 (1785)	61 (1669)	46 (6068)
Mucinous carcinoma	6 (212)	5 (162)	3 (117)	3 (86)	4 (577)
Endometrioid carcinoma	9 (331)	6 (216)	4 (151)	4 (99)	6 (797)
Clear cell carcinoma	5 (178)	4 (136)	4 (124)	3 (79)	4 (517)
Carcinosarcoma	3 (96)	3 (106)	3 (108)	2 (66)	3 (376)
Other/ Unspecified	33 (1198)	31 (1036)	27 (952)	21 (570)	28 (3756)
**Tumour grade, % per carcinoma type (n)**					
Serous carcinoma					
Grade 1	4 (50)	3 (37)	2 (28)	0.7 (11)	2 (126)
Grade 2	18 (225)	12 (171)	4 (63)	1 (24)	8 (483)
Grade 3	47 (574)	53 (737)	42 (753)	25 (409)	41 (2473)
Unknown	31 (385)	32 (435)	53 (941)	73 (1225)	49 (2986)
Mucinous carcinoma					
Grade 1	23 (49)	24 (39)	33 (39)	41 (35)	28 (162)
Grade 2	19 (40)	23 (37)	27 (32)	27 (23)	23 (132)
Grade 3	20 (43)	14 (22)	12 (14)	7 (6)	15 (85)
Unknown	38 (80)	40 (64)	27 (32)	26 (22)	34 (198)
Endometrioid carcinoma					
Grade 1	10 (34)	13 (28)	22 (33)	28 (28)	15 (123)
Grade 2	24 (79)	32 (69)	45 (68)	44 (44)	33 (260)
Grade 3	35 (116)	35 (75)	26 (40)	17 (17)	31 (248)
Unknown	31 (102)	20 (44)	7 (10)	10 (10)	21 (166)

Notes: Table shows column %. Grade is only shown for serous, mucinous, and endometrioid carcinomas, as clear cell carcinomas and carcinosarcomas are high grade by definition.

Supplementary Table 5: Additional 1, 5, and 10-year ovarian cancer survival, overall and by tumour stage at diagnosis and histological type, for women who survived the first year after ovarian cancer diagnosis

Ovarian cancer type	Cases	% survival at timepoint (95% CI)<br>1 year	 5 years	 10 years
All ovarian cancer	9807	79% (78-80)	46% (45-47)	37% (36-39)
Fully-malignant ovarian cancer (excluding borderline tumours)	8692	77% (76-77)	40% (39-41)	30% (29-31)
**Stage at diagnosis**				
Stage I	1797	98% (97-98)	88% (86-90)	82% (80-84)
Stage II	449	93% (90-95)	64% (59-69)	51% (45-56)
Stage III	2793	74% (72-76)	27% (25-29)	15% (13-16)
Stage IV	1052	63% (60-66)	17% (14-19)	9% (7-12)
Unknown	3716	77% (76-78)	46% (45-48)	39% (37-40)
**Ovarian cancer histotypes**				
Serous borderline tumour	507	99% (98-100)	95% (93-97)	93% (90-95)
Mucinous borderline tumour	608	99% (98-100)	98% (96-99)	97% (95-98)
Serous carcinoma	4845	76% (75-77)	32% (31-34)	21% (20-22)
Mucinous carcinoma	453	91% (88-94)	76% (71-80)	71% (66-75)
Endometrioid carcinoma	739	91% (89-93)	71% (68-75)	61% (57-65)
Clear cell carcinoma	438	81% (77-85)	61% (56-66)	54% (49-59)
Carcinosarcoma	222	65% (59-71)	32% (26-38)	27% (21-33)
Other/ Unspecified	1995	69% (67-71)	33% (31-35)	25% (23-27)

Note: Table shows ovarian cancer-specific survival at 1, 5, and 10 years after the first year from diagnosis, calculated using a lifetable approach, for all Million Women Study participants diagnosed with ovarian cancer, after exclusion of 3415 cases who died within the first year (N=9807). The above crude survival data is unadjusted for age, stage, or other factors.

Supplementary Figures

Supplementary Figure 1: Survival by time since diagnosis of ovarian cancer

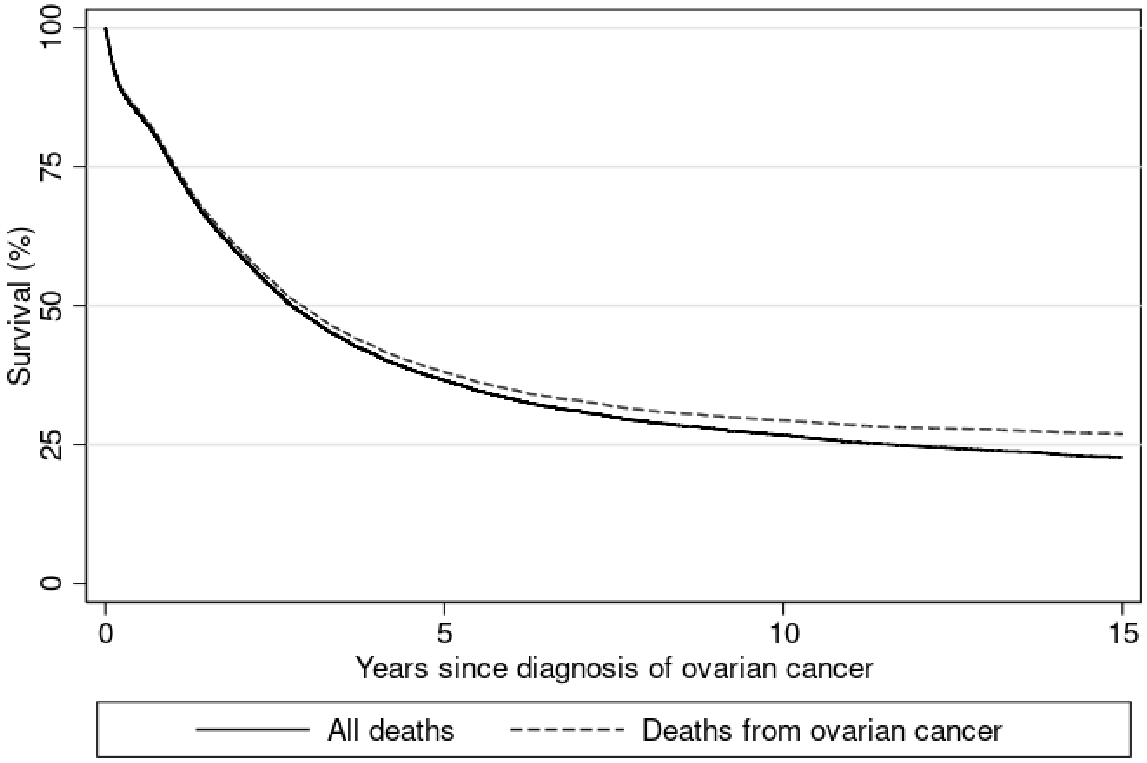

Note: This figure shows the Kaplan-Meier survival curves for survival after diagnosis of ovarian cancer, for all deaths, and for deaths attributed to ovarian cancer, for all Million Women Study participants diagnosed with ovarian cancer, after exclusion of 137 cases diagnosed at death (N= 13,085).

Supplementary Figure 2: Ovarian cancer survival by time and histological type in cases diagnosed at (A) Stage I and II and (B) Stage III and IV

(A)

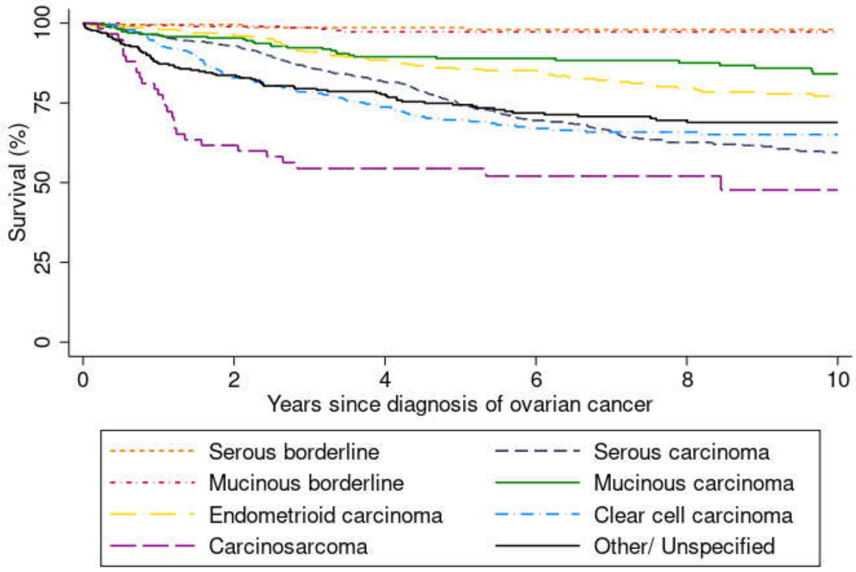

(B)

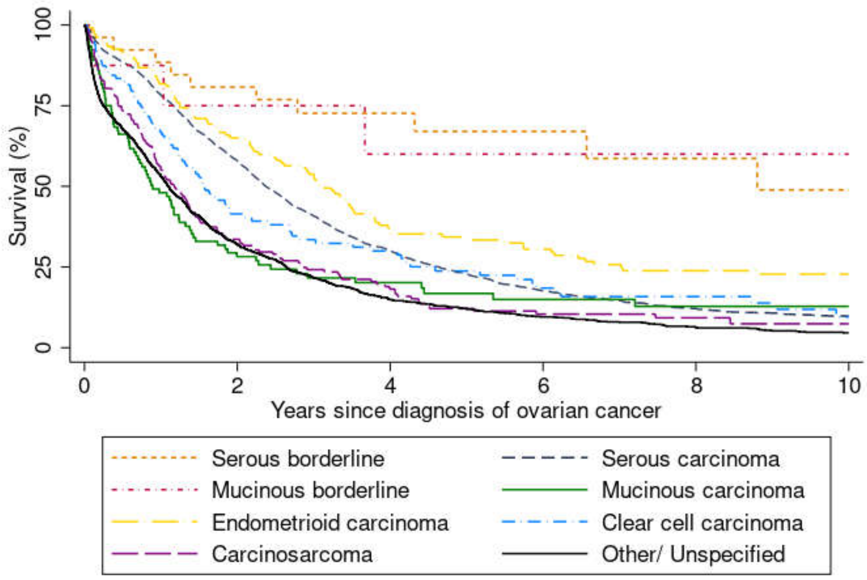

The figure shows the Kaplan-Meier survival curves for survival after diagnosis of ovarian cancer, for deaths attributed to ovarian cancer, by tumour histotype in (A) women diagnosed at Stage I and II and (B) women diagnosed at Stage III and IV, in women with ovarian cancer of known stage (N=7831).

Supplementary Figure 3: The association between BMI (per 5-unit increase) and ovarian cancer survival, by tumour histological type

Ovarian cancer: Histological type	Cases	Deaths	RR (95% CI)
Serous carcinoma	4047	2794	1.06 (1.01, 1.12)
Mucinous carcinoma	332	102	1.26 (0.94, 1.69)
Endometrioid carcinoma	456	157	1.03 (0.83, 1.29)
Clear cell carcinoma	335	153	1.10 (0.86, 1.41)
Carcinosarcoma	211	160	1.12 (0.86, 1.46)
Other/ unspecified	1505	1203	1.05 (0.97, 1.13)
**All ovarian cancer**	**6886**	**4569**	**1.06 (1.02, 1.11)**

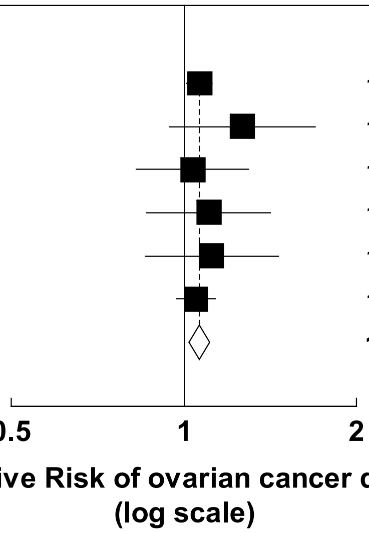

The figure shows the association between body mass index (BMI, per 5-unit increase) and risk of death from ovarian cancer, by tumour histological type, in Million Women Study participants with invasive ovarian cancer of known stage and with known BMI (n=6886). Likelihood ratio test for heterogeneity by histotype: p = 0.9.

Supplementary Figure 4: The association between smoking and ovarian cancer survival, by tumour histological type

(A) FORMER VS NEVER SMOKERS

Ovarian cancer: Histological type	Cases Former/Never	Deaths Former/Never	RR (95% CI)
Serous carcinoma	1125 / 2219	753 / 1549	0.95 (0.87, 1.04)
Mucinous carcinoma	76 / 167	27 / 50	1.14 (0.60, 2.18)
Endometrioid carcinoma	128 / 240	49 / 78	0.98 (0.90, 1.08)
Clear cell carcinoma	91 / 182	43 / 86	0.95 (0.52, 1.75)
Carcinosarcoma	56 / 106	40 / 86	0.94 (0.86, 1.03)
Other/ unspecified	405 / 796	311 / 633	0.76 (0.62, 0.93)
**All ovarian cancer**	**1881 / 3710**	**1223 / 2482**	**0.95 (0.88, 1.02)**

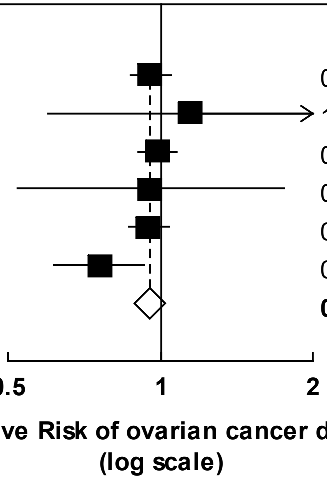

(B) CURRENT VS NEVER SMOKERS

Ovarian cancer: Histological type	Cases Current/Never	Deaths Current/Never	RR (95% CI)
Serous carcinoma	640 / 2219	464 / 1549	1.10 (0.98, 1.22)
Mucinous carcinoma	94 / 167	27 / 50	1.10 (0.98, 1.22)
Endometrioid carcinoma	80 / 240	29 / 78	1.27 (0.74, 2.19)
Clear cell carcinoma	52 / 182	22 / 86	0.99 (0.89, 1.10)
Carcinosarcoma	45 / 106	34 / 86	1.19 (0.66, 2.14)
Other/ unspecified	290 796	248 / 633	1.10 (0.98, 1.22)
**All ovarian cancer**	**1201 / 3710**	**824 / 2482**	**1.17 (1.07, 1.27)**

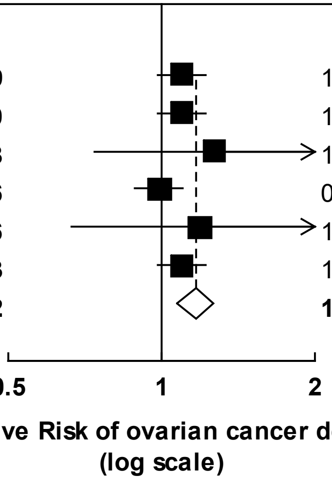

The figure shows the association between smoking (former smokers vs never smokers (A), and current smokers vs never smokers (B)) and risk of death from ovarian cancer, by tumour histological type, in Million Women Study participants with invasive ovarian cancer of known stage and with known smoking history (n=6792). Likelihood ratio test for heterogeneity by histotype: p = 0.6.
